# Evidence of drought memory in *Dipteryx alata* indicates differential acclimation of plants to savanna conditions

**DOI:** 10.1038/s41598-020-73423-3

**Published:** 2020-10-05

**Authors:** Rauander D. F. B. Alves, Paulo E. Menezes-Silva, Leticia F. Sousa, Lucas Loram-Lourenço, Maria L. F. Silva, Sabrina E. S. Almeida, Fabiano G. Silva, Leonardo Perez de Souza, Alisdair R. Fernie, Fernanda S. Farnese

**Affiliations:** 1 Instituto Federal Goiano, Campus Rio Verde, Rio Verde, GO 75.901-970 Brazil; 2 Max-Planck-Institute for Molecular Plant Physiology, 14476 Potsdam-Gölm, Germany

**Keywords:** Biochemistry, Chemical biology, Plant sciences

## Abstract

The remarkable phytogeographic characteristics of the Brazilian savanna (Cerrado) resulted in a vegetation domain composed of plants with high structural and functional diversity to tolerate climate extremes. Here we used a key Cerrado species (*Dipteryx alata*) to evaluate if species of this domain present a mechanism of stress memory, responding more quickly and efficiently when exposed to recurrent drought episodes. The exposure of *D. alata* seedlings to drought resulted in several changes, mainly in physiological and biochemical traits, and these changes differed substantially when the water deficit was imposed as an isolated event or when the plants were subjected to drought cycles, suggesting the existence of a drought memory mechanism. Plants submitted to recurrent drought events were able to maintain essential processes for plant survival when compared to those submitted to drought for the first time. This differential acclimation to drought was the result of orchestrated changes in several metabolic pathways, involving differential carbon allocation for defense responses and the reprogramming and coordination of primary, secondary and antioxidant metabolism. The stress memory in *D. alata* is probably linked the evolutionary history of the species and reflects the environment in which it evolved.

## Introduction

Considered a global biodiversity hotspot with a high degree of endemism^1^, the Brazilian savanna (Cerrado) is composed by a mosaic of plant formations that occupy about 21% of the Brazilian territory and harbors amongst the greatest floral diversity on the planet^2^. To understand the flora of this domain, it is important to consider the forest refuges theory^3^, which predicts the existence of speciation in forest refuges formed as a result of forest expansion and retraction during glaciation and deglaciation periods that occurred during the quaternary. Studies of the Brazilian phytogeography demonstrated that the Cerrado may have been a transition zone in which species were exposed to fluctuating environmental conditions for thousands of years^4^, resulting in the emergence of the high structural and functional diversity in morpho-anatomical and physiological traits which allows species of this domain to tolerate contrasting climatic extremes. An example of such fluctuation in environmental conditions, commonly observed in Cerrado, is its well-defined seasons, characterized by a long period of water restriction (approximately five months), followed by a rainy period^5^. In this regard, the plant species that inhabit this domain tend to be naturally acclimated to periodic drought events, being good models for the study of adaptation mechanisms to water scarcity.

Drought is the main environmental factor limiting the growth, distribution, and survival of plant species worldwide^6^. The damages triggered by water restriction are diverse and often result in the reduction of CO_2_ availability for the photosynthetic process due to stomatal closure to prevent excessive water loss through transpiration^7^. Moreover, drought can result in the generation of reactive oxygen species (ROS), which can cause severe damage to the cellular machinery^8^. It should be noted, however, that the deleterious effects of drought on plant performance and survival can be mitigated through the activation of different defense mechanisms, such as morpho-anatomical changes in different organs to modulate water uptake and transport, and biochemical and physiological changes, such as the strict control of stomatal closure, expression of antioxidant enzymes and production of osmocompatible solutes^9^.

Exposure time, stress intensity and the occurrence of more than one stressful event are factors that interfere in plant responses to drought^10^. However, although important advances have been made in the comprehension of the physiological mechanisms underlying the acclimation to drought under different scenarios of intensity and duration, the majority of studies still analyze this stress as an isolated event^11–13^. This is despite the fact that it is well known that, in many environments, such as the Brazilian Cerrado and other savannas, the same plant goes through several drought cycles during its growth and development^14^. Some authors have already demonstrated that a pre-exposure to the stressor can alter subsequent responses of the plant, which may result in increased sensitivity^15,16^, or even in greater tolerance^17^ to subsequent stressful events. In this regard, studies with plants subjected to repeated stressful events demonstrate the existence of a differential plant acclimation mechanism, also called “stress memory”^18^.

Stress memory can be defined as the set of structural, genetic and biochemical modifications that occur as a result of the recurrent stress exposition^19^, being considered short-term (less than a week), which is hardly passed on to future generations, or long-term, where the alterations must exceed the event that caused it and activate a faster and/or stronger response in the face of a new stress event^20^. The existence of an acclimation process through long-term memory has a great ecological impact since it can reflect the evolutionary history of a species, which, in turn, results in a greater or lesser response capacity by plants facing climatic fluctuations^21,22^. The lack of this capacity, or even the loss of resilience after recurring stressful events, may indicate serious environmental constrains in a climate change scenario, triggering the replacement of species or even entire biomes^15^. Thus, it is important that studies which aim to characterize plant responses to water deficit work with repeated cycles of drought and the recovery time of these cycles should be greater than short-term memory. It should also be noted that although the existence of differential acclimation has already been characterized in some species subjected to drought cycles, the few existing studies have been performed predominantly with clones and/or commercial cultivars^23–25^. Given these facts, there is currently little information available about the existence of stress memory in native plants from environments where drought occurs naturally in a cyclical manner. Thus, the possible role of differential acclimation to stress in the evolutionary history of species inhabiting those sites is not clear. To fill this gap, the main objective of the present study was to evaluate the existence of drought memory in seedlings of *Dipteryx alata*, a perennial native Cerrado species. This species plays pivotal roles in the conservation of this domain, due to its high germination rates, seedling establishment and production of fresh pulp during the dry season, being fundamental for feeding the local fauna during this period^26^. In addition, *D. alata* has a high success rate in the Cerrado environment when considering the rate of survival, growth, and reproduction. In fact, studies of recovery of Cerrado, in which typical species from this domain were planted to recover degraded areas, have observed that *D. alata* is the plant that has the highest survival rate ten years after planting (96% survival rate)^27^, the one with the highest growth rate after 26 months of planting^28^ and also one of the few species that is able to reproduce during the dry season^26^. Seedlings of this species were subjected to repeated drought cycles to test the following three hypothesis: (i)* D. alata* presents differential stress acclimation, being able to present more robust response mechanisms after recurrent drought cycles; (ii) The differential stress acclimation in *D. alata* is related to biochemical and physiological alterations; and (iii) Plants subjected to a single drought cycle are more sensitive to stress, showing greater cellular and physiological damage.

## Results

### Water relations

**Figure 1:**
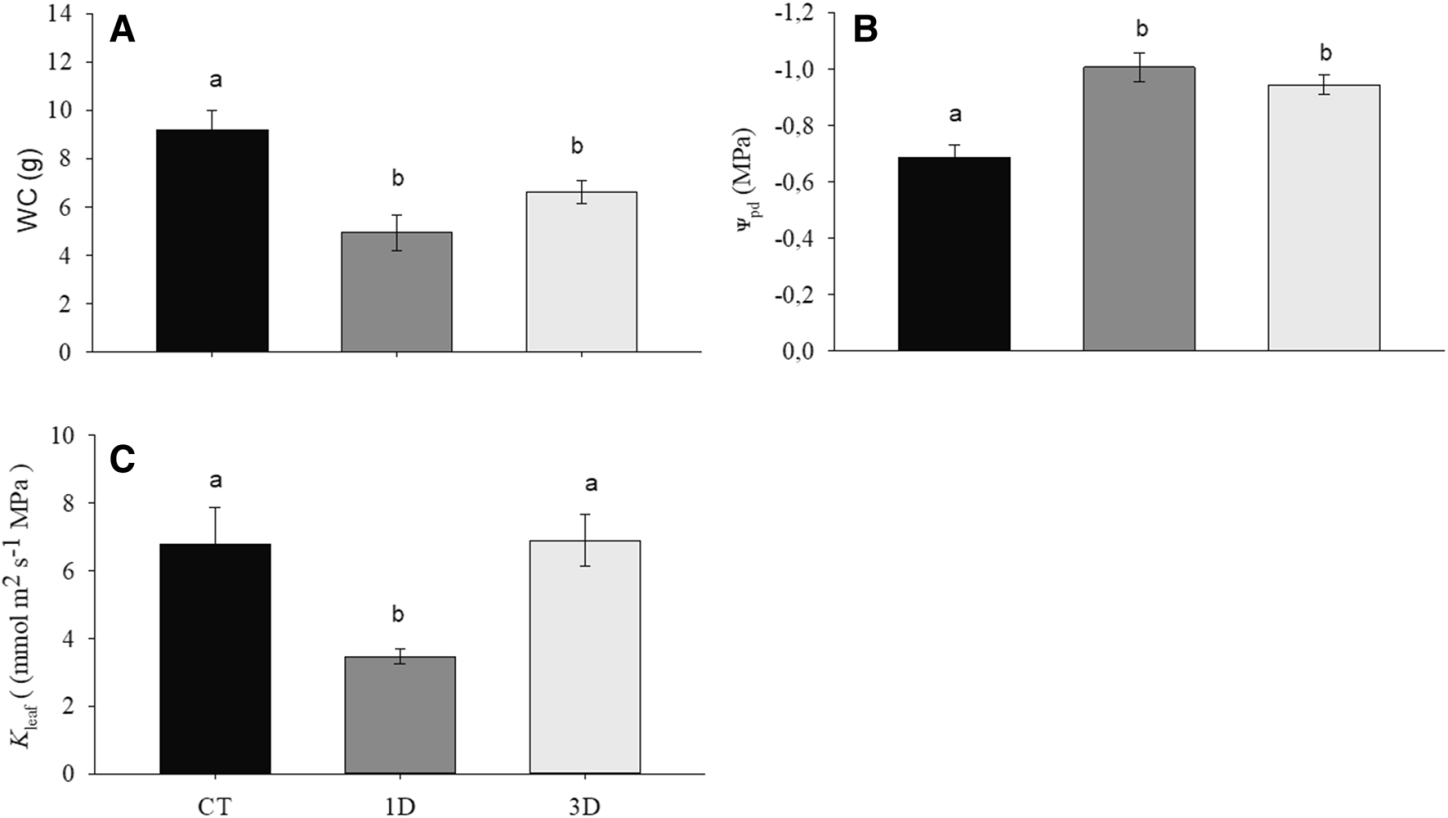
Water content (WC) (**A**), predawn water potential (Ѱ_pd_) (**B**) and leaf hydraulic conductivity (*K*_leaf_) (**C**) in *Dipteryx alata* seedlings exposed to continuous irrigation (control, CT), one drought cycle (1D) and three drought cycles (3D) for 6 days. Means followed by the same letter do not differ from each other by the SNK test (P ≤ 0.05).

All results presented in this section and in the following ones were collected on the last day of application of the treatments, that is, after six days of exposure of the plants to one or three cycles of drought. The water restriction changed the leaf water content (WC) of the treatments, with highest values in the control and the lowest values in plants exposed to drought treatments (Fig. 1A). The same pattern was observed in relation to predawn water potential (Ѱ_pd_) (Fig. 1B). Leaf hydraulic conductivity (*K*_leaf_), in turn, decreased only in plants subjected to a single drought event (Fig. 1C).

### Morphological and anatomical traits

**Figure 2:**
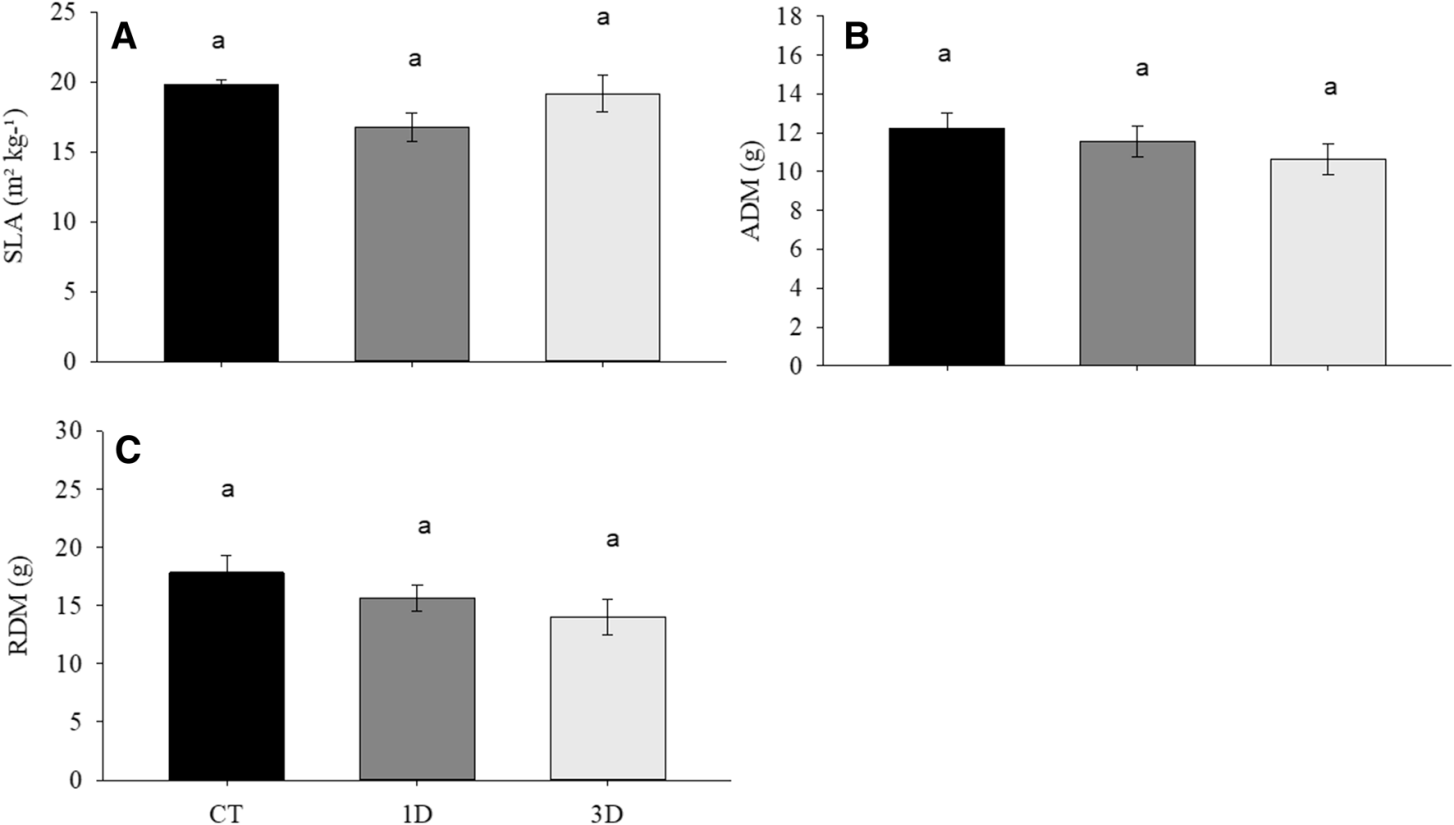
Specific leaf area (SLA) (**A**), aerial dry mass (ADM) (**B**) and root dry mass (RDM) (**C**) in *Dipteryx alata* seedlings exposed to continuous irrigation (control, CT), one drought cycle (1D) and three drought cycles (3D) for 6 days. Means followed by the same letter do not differ from each other by the SNK test (P ≤ 0.05).

**Figure 3:**
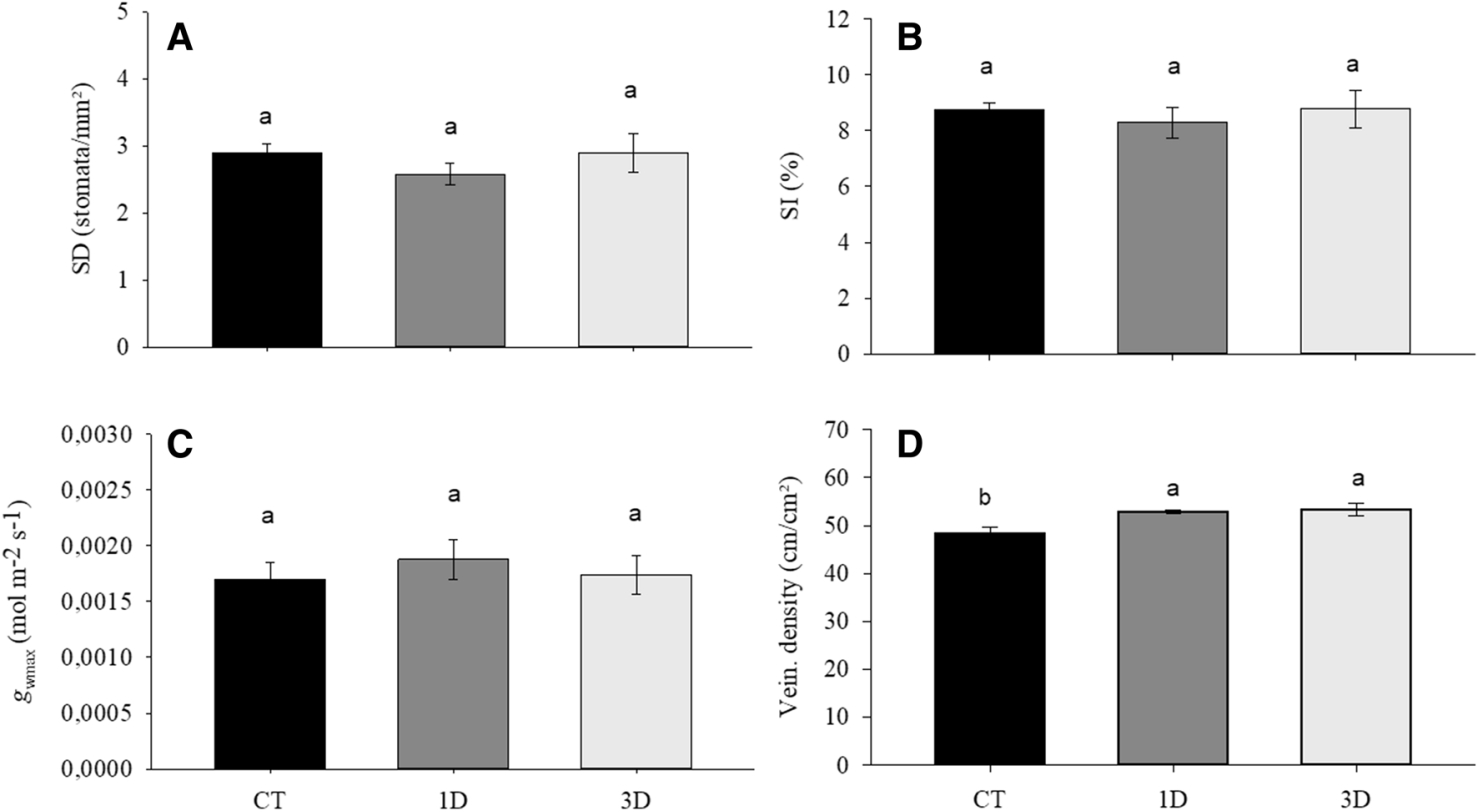
Stomatal density (SD) (**A**), stomatal index (SI) (**B**), maximum stomatal conductance (*g*_wmax_) (**C**) and vein density (VD) (**D**) in *Dipteryx alata* seedlings exposed to continuous irrigation (control, CT), one drought cycle (1D) and three drought cycles (3D) for 6 days. Means followed by the same letter do not differ from each other by the SNK test (P ≤ 0.05).

The exposure of *D. alata* seedlings to drought conditions did not alter their biomass production (Fig. 2A–C). Similarly, no significant differences were observed for most of the anatomical traits analyzed between any of the treatments (Fig. 3A–C). The only exception to this was the increase in vein density in both drought treatments when compared to CT plants, but with no significant differences between them (Fig. 3D).

### Physiological traits

**Figure 4:**
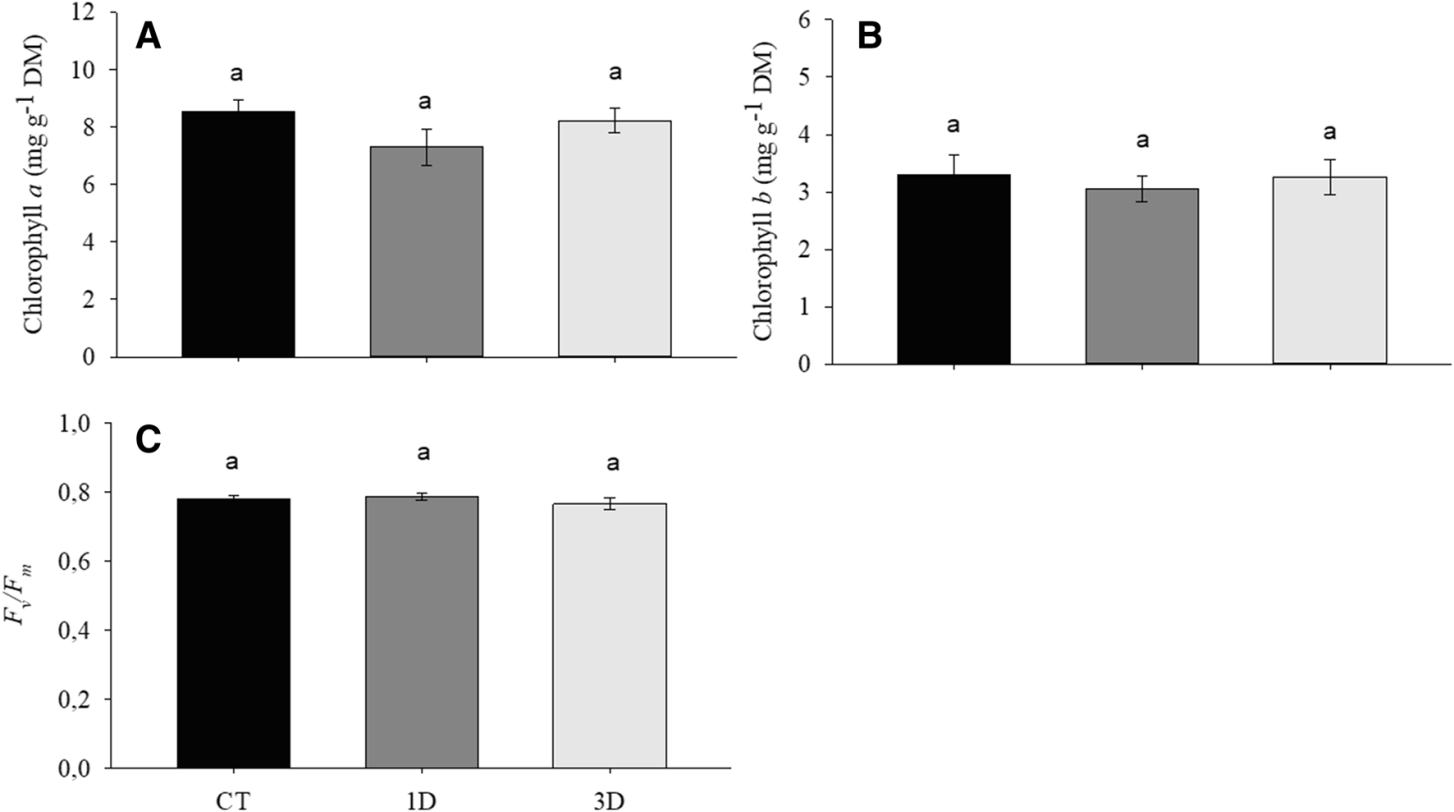
Chlorophyll *a* (**A**), chlorophyll *b* (**B**) and potential quantum yield of photosystem II (*F*_v_/*F*_m_) (**C**) in *Dipteryx alata* seedlings exposed to continuous irrigation (control, CT), one drought cycle (1D) and three drought cycles (3D) for 6 days. Means followed by the same letter do not differ from each other by the SNK test (P ≤ 0.05).

**Figure 5:**
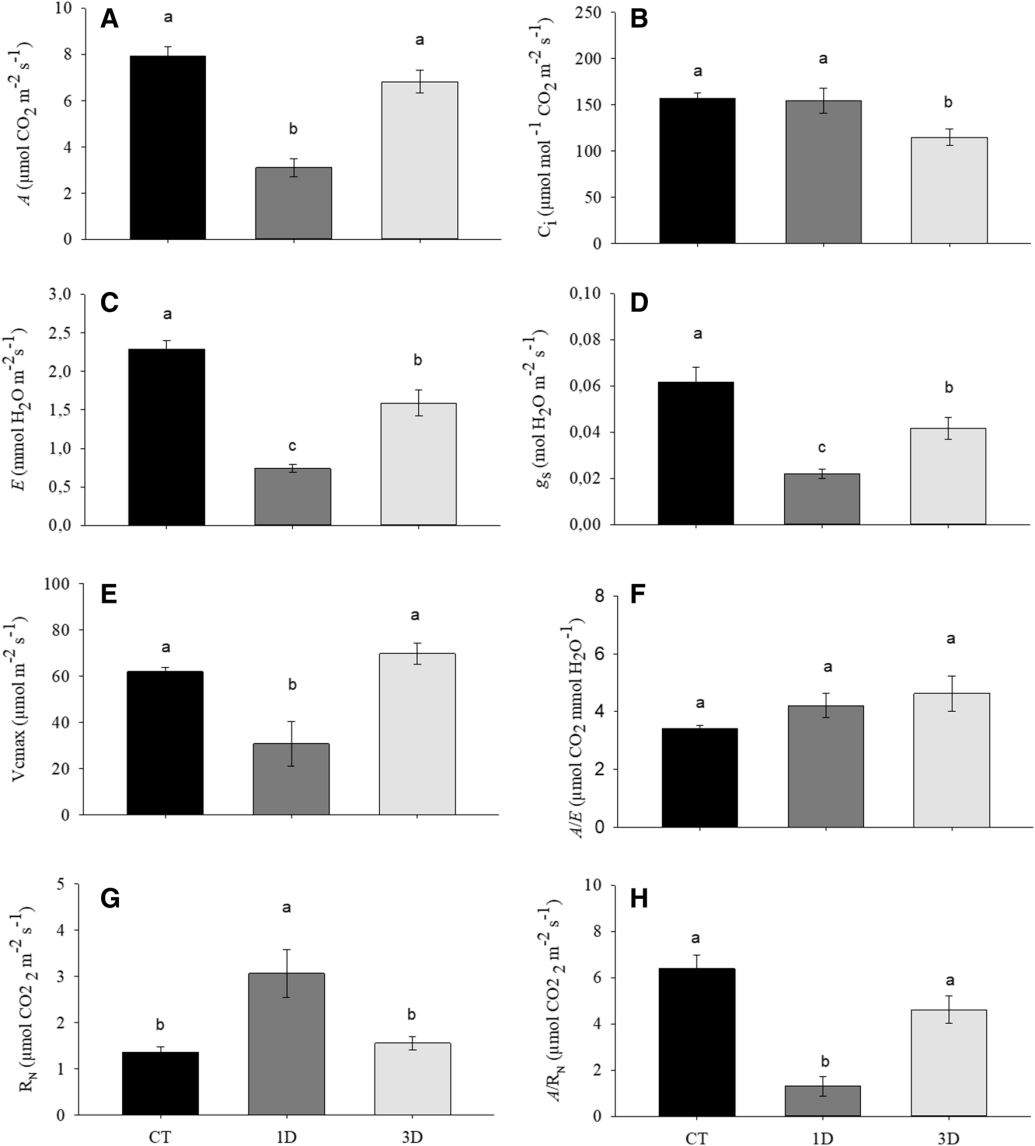
Net carbon assimilation rate (*A*) (**A**), internal CO_2_ concentration (Ci) (**B**), transpiration rate (*E*) (**C**), stomatal conductance (*g*_s_) (D), Rubisco maximum carboxylation rate (V_cmax_) (**E**), water use efficiency (*A*/*E*) (**F**), dark respiration (R_N_) (**G**) and *A*/R_N_ ratio (**H**) in *Dipteryx alata* seedlings exposed to continuous irrigation (control, CT), one drought cycle (1D) and three drought cycles (3D) for 6 days. Means followed by the same letter do not differ from each other by the SNK test (P ≤ 0.05).

Regardless of treatments, no significant changes were observed in the levels of chlorophylls *a* and *b* or the potential quantum yield of photosystem II (*F*_v_*/F*_m_) (Fig. 4A–C). Gas exchange traits, on the other hand, were deeply affected by water deficit. The net rate of carbon assimilation (*A*), for example, was similar in CT and 3D plants, but showed a sharp decrease in 1D plants (Fig. 5A), while the internal concentration of CO_2_ (C_i_) decreased only in 3D plants (Fig. 5B). The transpiration rate (*E*) varied significantly between all the treatments, with the highest values found in CT and the lowest in 1D plants (Fig. 5C), reflecting the values observed for stomatal conductance (*g*_*s*_) (Fig. 5D). Regarding the maximum rate of carboxylation of Rubisco (V_cmax_), significant decreases were observed only in plants exposed to a single drought event (Fig. 5E). Finally, the water use efficiency (*A*/*E*) (Fig. 5F) was invariant between treatments.

Besides the changes observed in the photosynthetic traits, dark respiration (R_N_) also changed in response to water restriction, increasing considerably in 1D plants (Fig. 5G). In fact, in this treatment, respiration was more than two-fold higher than in CT or 3D plants. Consequently, the *A*/R_N_ ratio was much lower when the drought was imposed as a single event (Fig. 5H).

### Cell damage

**Figure 6:**
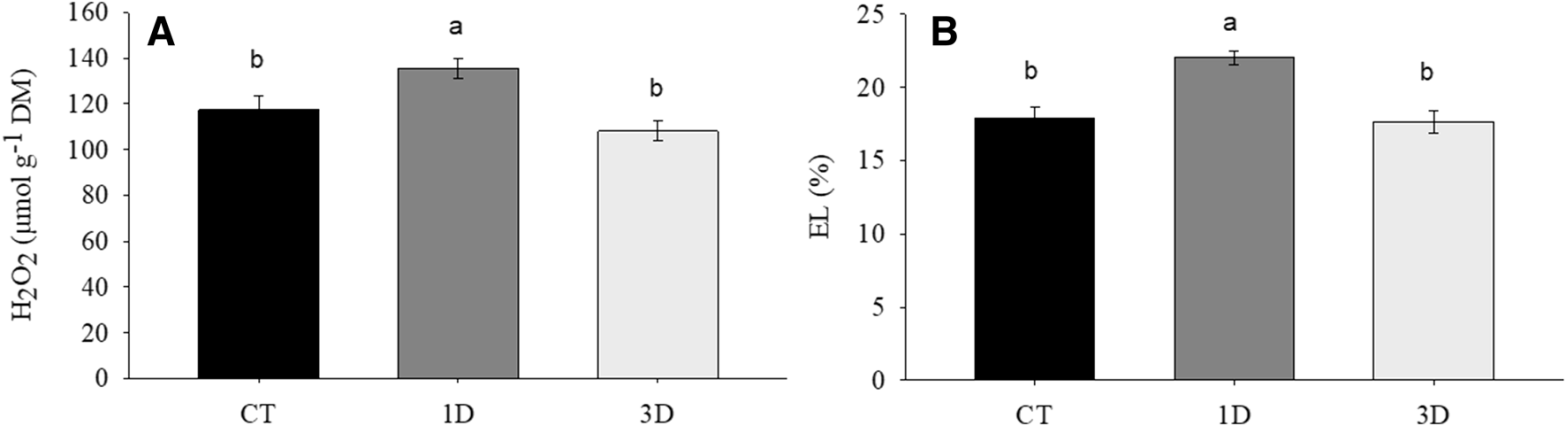
Hydrogen peroxide concentration (H_2_O_2_) (**A**) and Electrolyte leakage (EL) (**B**) in *Dipteryx alata* seedlings exposed to continuous irrigation (control, CT), one drought cycle (1D) and three drought cycles (3D) for 6 days. Means followed by the same letter do not differ from each other by the SNK test (P ≤ 0.05).

*Dipteryx alata* exposure to a single drought event increased the generation of reactive oxygen species, as evidenced by the increase in hydrogen peroxide concentration (Fig. 6A), leading to damages to cell membranes, denoted by the increased electrolyte leakage (Fig. 6B). It is interesting to note, however, that when the plants were subjected to three drought cycles, these damages were attenuated, and the levels of hydrogen peroxide and electrolyte leakage remained similar to those observed in CT plants.

### Enzymes of the antioxidative metabolism

**Figure 7:**
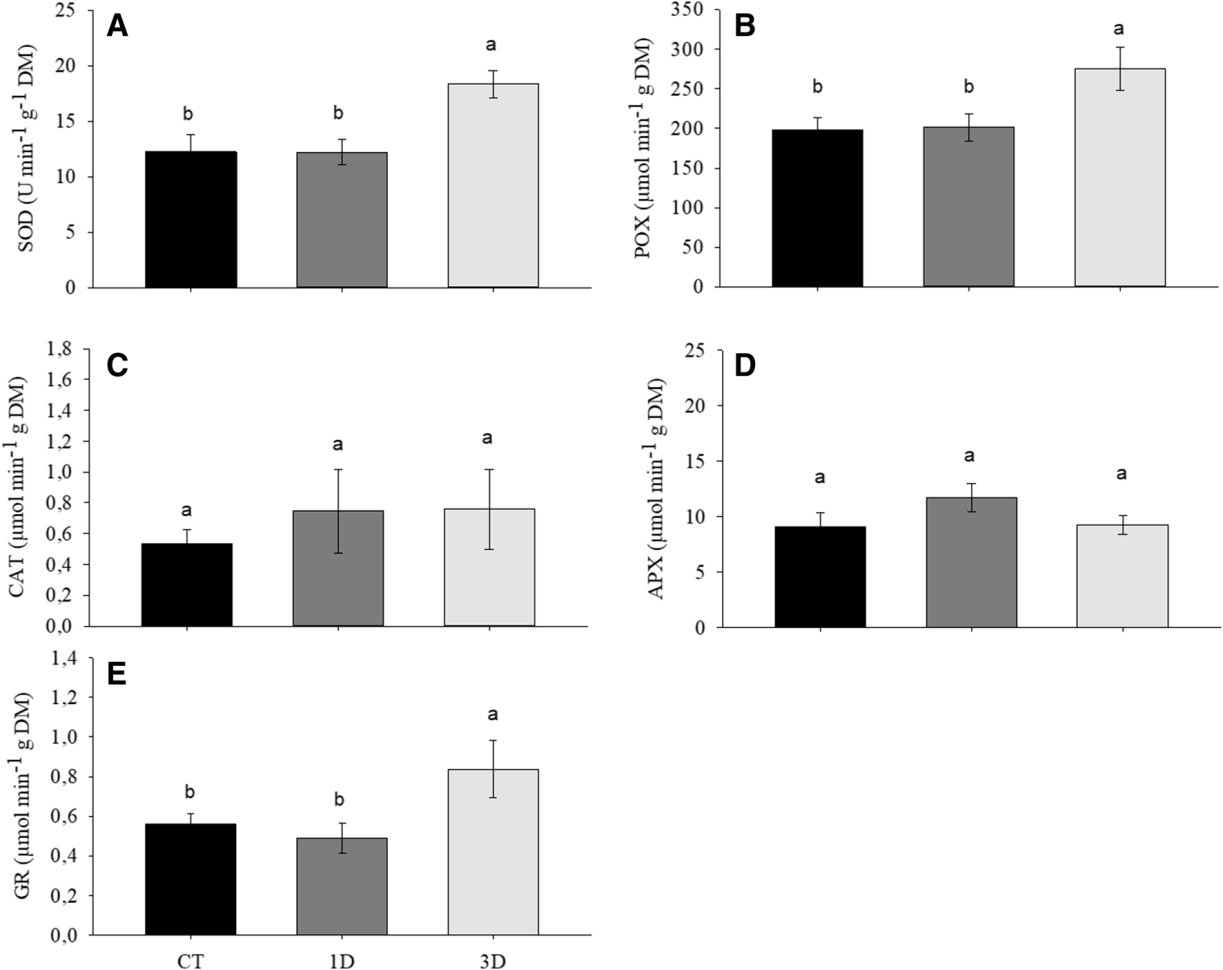
Enzymatic antioxidant system: superoxide dismutase (SOD) (**A**), peroxidase (POX) (**B**), catalase (CAT) (**C**), ascorbate peroxidase (APX) (**D**) and glutathione reductase (GR) in *Dipteryx alata* seedlings exposed to continuous irrigation (control, CT), one drought cycle (1D) and three drought cycles (3D) for 6 days. Means followed by the same letter do not differ from each other by the SNK test (P ≤ 0.05).

Exposure to a single drought event was not sufficient to activate antioxidant metabolism in *D. alata* seedlings. On the other hand, when these plants were exposed to repeated drought cycles, there was a substantial increase in the activity of most enzymes (SOD, POX, and GR). CAT and APX were not responsive to any of the treatments (Fig. 7A–E).

### Metabolic profile

**Figure 8:**
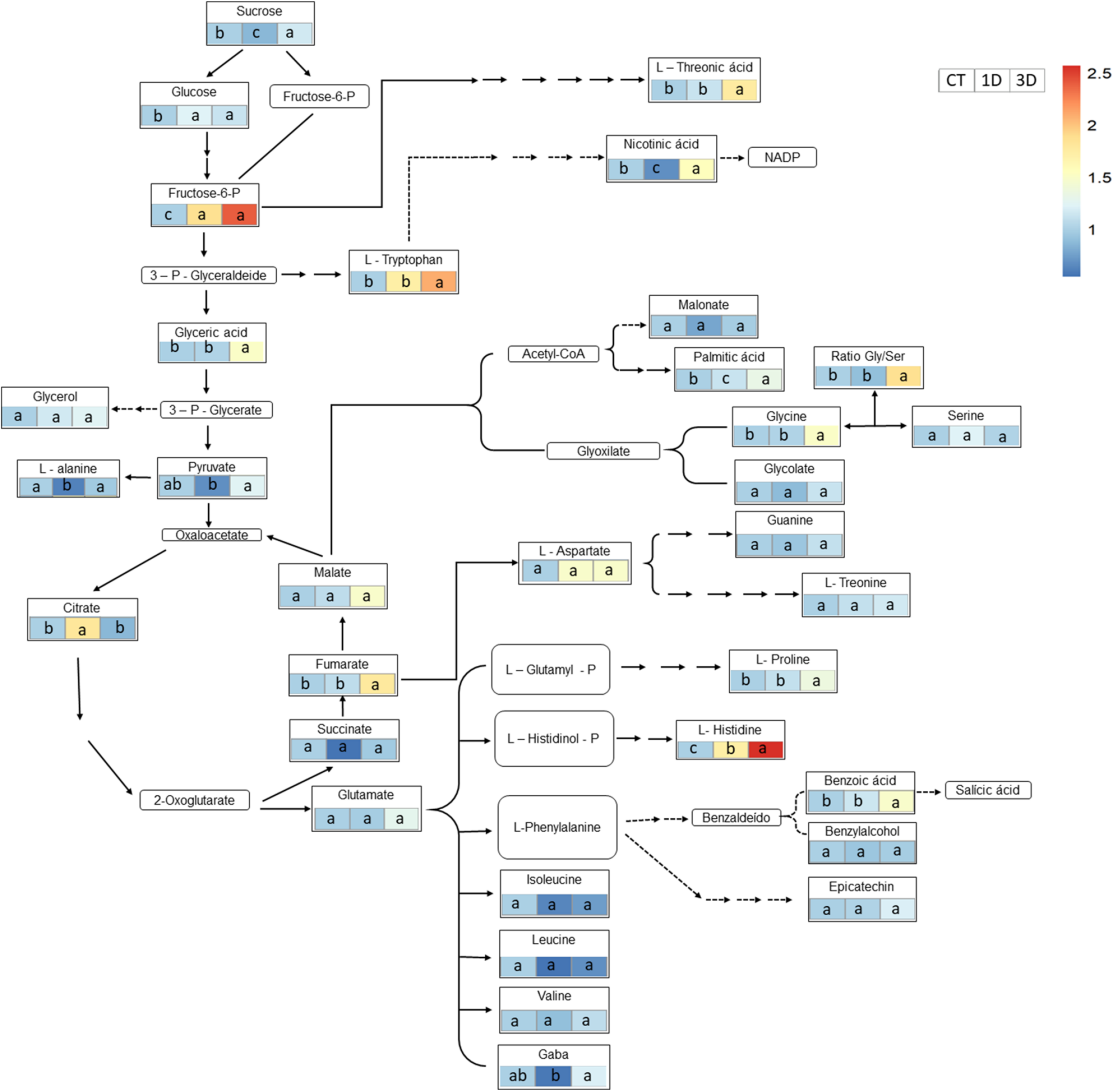
Major metabolic alterations in *Dipteryx alata* seedlings exposed to continuous irrigation (control, CT), one drought cycle (1D) and three drought cycles (3D) for 6 days. Values are the fold change relative to control mean. Boxes followed by the same letter do not differ from each other by the SNK test (P ≤ 0.05).

**Figure 9:**
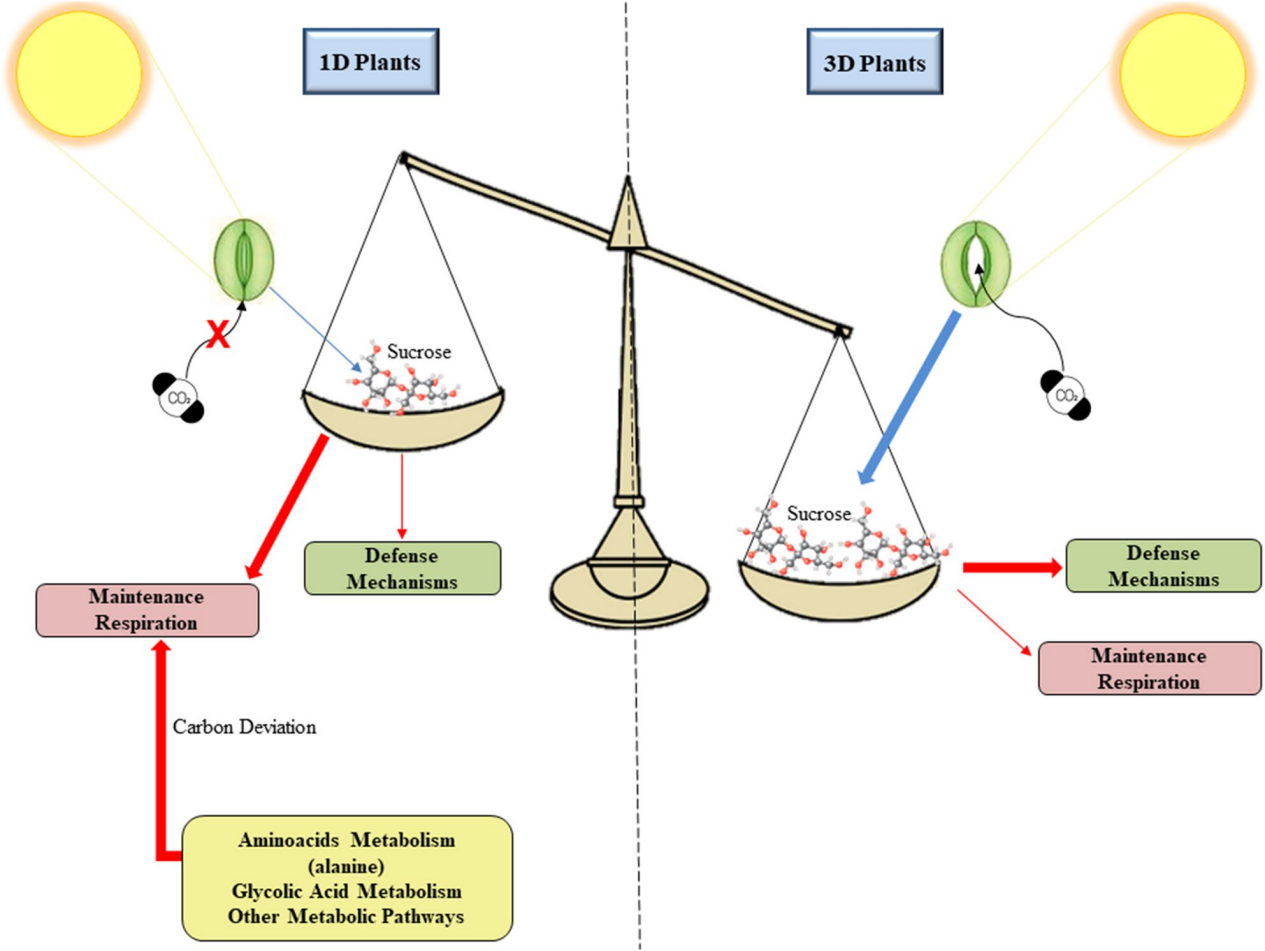
Carbon balance in plants exposed to 1 (1D plants) or 3 (3D plants) drought cycles. Blue arrows indicate anabolic process (photosynthesis) and red arrows indicate catabolic processes. The thickness of the arrow indicates the intensity of the process. Plants that experienced 3 drought cycles were able to keep their stomata open and, consequently, had a higher rate of carbon fixation, which was directed to defense mechanisms. Activation of defense responses maintained cell integrity, which allowed maintenance respiration to continue at levels similar to control. In plants exposed to a single drought cycle, stomatal closure resulted in less carbohydrate production, which associated with less activation of defense mechanisms triggered several cellular damages. The high increase in maintenance respiration in these plants required the deviation of metabolites from other pathways to the TCA cycle.

The analysis of the metabolic profile showed important biochemical changes in response to drought stress, with more than half of the analyzed metabolites showing a statistical difference in at least one of the treatments (Figs. 8, 9). Most changes occurred in plants exposed to repeated drought cycles and these changes demonstrated alterations in both primary and secondary metabolism. Regarding primary metabolism, the main adjustments were observed in the levels of sugars (fructose, sucrose, and glucose), organic acids (citrate, fumarate, pyruvate, malate, and succinate) and amino acids. Sucrose and fructose contents increased only in 3D plants, while glucose levels increased in both drought treatments. Regarding organic acids, the levels of pyruvate were lower in 1D than in 3D plants, while the citrate content was much higher in plants exposed to a single drought event. Fumarate, threonic acid and palmitic acid, increased only in response to successive drought cycles. Amino acids represent another class of molecules that showed contrasting levels between treatments, with variations in the content of alanine, GABA, glycine, histidine, tryptophan and proline being higher in plants exposed to three drought cycles compared to those exposed to just a single stress event. Recurrent drought events also lead to increased apparent glycine/serine ratio, a result not observed for 1D plants. Finally, 3D plants showed an increase in the levels of nicotinic and benzoic acids, in relation to 1D plants, suggesting a higher deviation of molecules from primary to secondary metabolism.

### Relationship between variables

**Figure 10:**
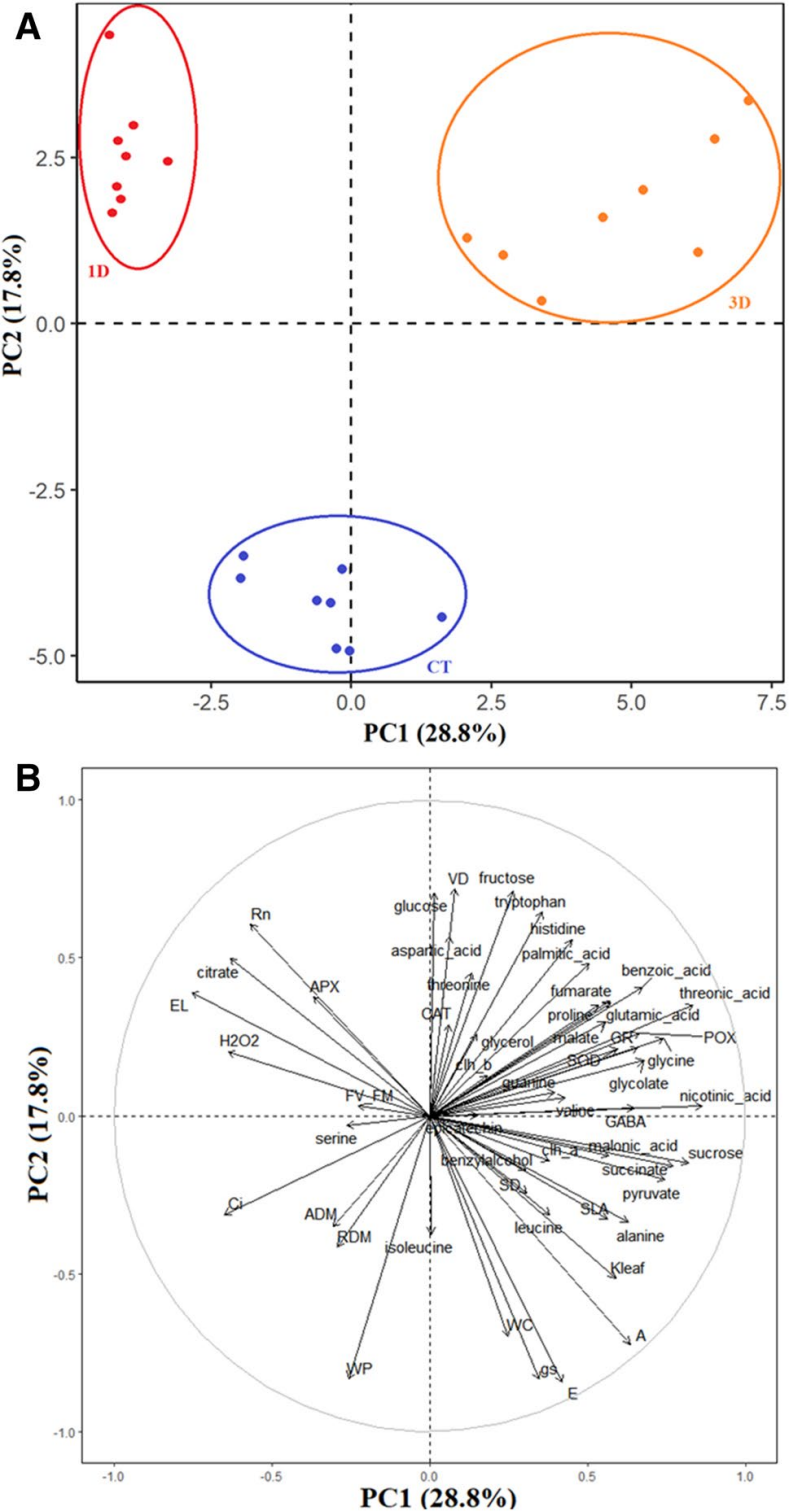
Multivariate analysis (PCA analysis). Two-dimensional PCA biplots showing associations between experimental groups and analysis spots generated by principal component analysis (PCA). The segregation of the experimental groups (**A**) and the correlation coefficients for all the analysis (**B**) were plotted in the first two component spaces.

The first two PCs explained 50.7% of the observed variation (PC1 = 28.8%; PC2 = 17.8%; Fig. 10), with a clear separation observed between CT, 1D and 3D plants. The separation between treatments shows that each growth condition resulted in distinct biochemical and physiological responses so that in each condition the plants represented a functional group with differential metabolic characteristics. In fact, while cell damage (electrolyte leakage and H_2_O_2_ concentration) and cell respiration traits contributed to the separation of plants exposed to only one stressful event, antioxidant defense mechanisms and metabolites related to secondary metabolism and osmotic adjustment were more strongly linked to plants exposed to three drought cycles.

## Discussion

Natural environments are dynamic ecosystems in which plants are often subjected to stressful events that occur cyclically throughout their life. In this context, differential acclimation represents a crucial factor to be considered in order to better understand the ecology and evolutionary history of plant species, with potential implications in conservation programs. Despite the relevance of this kind of information, only recently this aspect has started to be evaluated on drought studies, and the data available so far have focused predominantly on clones and/or commercial crops^23,25^. In the present study, the differential acclimation to drought was further investigated in seedlings of *Dipteryx alata*, a native Cerrado species which, in natural conditions, is recurrently submitted to variations in water availability. The exposure of *D. alata* seedlings to drought resulted in several changes, mainly in physiological and biochemical traits, and these changes differed substantially when the water deficit was imposed as an isolated event or when the plants were subjected to drought cycles. In general, the results obtained in the present study suggest the existence of a differential acclimation mechanism responsible for a faster and more effective response to the cyclic stressful event in *D. alata*.

### Differential acclimation of the photosynthetic apparatus in plants exposed to drought cycles

Photosynthesis plays a central role in plant metabolism, being a key trait for assessing plant fitness. Under drought conditions, there are three main limitations to the photosynthetic process: (i) diffusive; (ii) hydraulic; and (iii) biochemical limitations^29^. Although there were no changes in the pigment content or *F*_v_/*F*_m_ (Fig. 4A–C), our results indicate that the three factors (diffusive, hydraulic and biochemical) were involved in the lower carbon assimilation in the plants exposed to a single drought event while 3D plants were able to maintain photosynthetic performance similar to CT plants (Fig. 5A).

The diffusive limitation to photosynthetic is mainly determined by the stomatal (*g*_s_) and mesophyll (*g*_m_) resistances that CO_2_ encounters during its diffusion^30^. Although a decrease in *g*_s_ was observed in both drought treatments, this decrease was much more pronounced in 1D plants (Fig. 5D). It should be noted that *g*_m_ is intrinsically co-regulated with *g*_s_^31^ and it is therefore feasible to suggest that both stomatal and mesophyll limitations were involved in the decreased CO_2_ assimilation in 1D plants. Another factor strictly related to *g*_s_ rates concerns the plant’s ability to transport water from the roots to the transpiring tissues, since plants with greater rehydration capacity are able to maintain high *g*_s_^29,32^. Indeed, the coregulation of leaf hydraulics and *g*_s_ has been described as an important step in vascular plants evolution to maximize photosynthesis^29^. Therefore, the drop in leaf hydraulic conductivity (*K*_leaf_) in 1D plants (Fig. 1C) was also a major factor in reducing their photosynthetic performance^33^.

In addition to the diffusive and hydraulic limitations, under more severe water deficit conditions, reductions in photosynthetic rates may also occur due to the inhibition of specific metabolic processes, such as a reduction in the Calvin–Benson cycle enzymatic activity (biochemical limitations)^34,35^. Despite the marked reduction in *g*_*s*_, 1D plants showed internal CO_2_ concentration values (*Ci*) similar to the control plants (Fig. 5B,D), indicating that not all CO_2_ entering the sub-stomatal chamber was being fixed by the Calvin–Benson cycle enzymes, which are sensitive to drought events, especially Rubisco^36^. This observation is consistent with the reduction in V_cmax_ on those plants (Fig. 5E), a trait that indicates Rubisco's carboxylation speed, suggesting the occurrence of impairment in the optimal activity of this enzyme in plants exposed to a single drought event^37^.

In direct contrast to what was observed for 1D plants, after the exposure to three drought cycles, the photosynthesis of *D. alata* plants was restored. One factor that may have contributed greatly to the recovery of photosynthetic capacity in 3D plants was the maintenance of *K*_leaf_ (Fig. 1C), which allowed the maintenance of greater stomatal opening compared to 1D plants. Since the three limiting factors to photosynthesis (diffusive, hydraulic and biochemical) were observed in 1D plants, the differential acclimation of the photosynthetic process to drought in the 3D plants required more than increments in *K*_leaf_ and *g*_s_, also involving the maintenance of the biochemical apparatus of photosynthesis. Indeed, the values of *C*_*i*_ and V_cmax_ in 3D plants indicate that there was no biochemical impairment of the photosynthetic process, suggesting that the observed differential acclimation required the occurrence of orchestrated alterations in different traits in order to maintain cellular homeostasis.

### Negative carbon balance impairs the activation of defense mechanisms

The behavior of plants subjected to a single drought event was characterized by increases in respiration and a suppression of photosynthesis, resulting in a negative carbon balance. This imbalance between use and absorption of light energy frequently occurs in plants after exposure to several types of stress^30,38^, and has been associated with an increased flux of carbon skeletons and ATP to repair damaged cell structures (maintanance respiration)^39^. Whilst in the short-term this represents a sensible defense strategy, it should be noted that in the long-term the increase in respiration could deplete the plant's reserves if it is not accompanied by gains in photosynthesis. In fact, it has already been demonstrated in some species exposed to drought that the increase in respiratory rate occurs only transiently in the initial stages of stress, decreasing afterward^30^.

The increment in respiration in 1D plants was followed by several changes in the levels of metabolites involved in this process (Fig. 8). The decrease in the levels of sucrose and pyruvate, observed only in 1D plants, may represent the exhaustion of these molecules due to intense sucrolysis aimed at providing enough substrate for the operation of the TCA cycle^30^. This evidence is reinforced by the large increase in the levels of citrate, the first substrate of the TCA cycle, formed from the oxidative decarboxylation of pyruvate. It is interesting to note, however, that the carbohydrate pool in 1D plants was not sufficient to supply the cell’s energy demands, which apparently required the deviation of carbon from process other than the respiratory pathways, with decreases in the levels of specific molecules such as alanine. The decrease in alanine levels in leaf tissues during drought events occurs due to its degradation to donate the amino group to form pyruvate through the action of the enzyme serine:glyoxylate aminotransferase^40^. Similarly, the lower levels of glycolic acid in 1D plants, compared to 3D plants, may be a consequence of the conversion of this metabolite to 3-phosphoglycerate, in order to maintain the flux of carbon skeletons into the respiratory pathways^41^. Alternatively, glycolic acid may have been metabolized to form amino acids and thereby supply the deficit generated by amino acids deviation to respiration^42^. The negative carbon balance in 1D plants, as well as the need for metabolic shifts with molecules deviation from other pathways to sustain the respiratory metabolism, resulted in less carbon for defense mechanisms, which can be evidenced, for example, by the decreased levels of secondary metabolites or by the absence of antioxidant system activation. The reduced capacity of 1D plants to activate cell defense systems likely lead to an increased occurrence of damage which would be anticipated to further compromise central physiological processes and overall plant fitness, when compared to 3D plants.

The negative carbon balance in 1D plants, represented in Fig. 9, involved a complex network of interconnected factors, which included reduction in carbon assimilation, increase in metabolic costs to repair damaged structures (maintenance respiration) and the targeting of carbon from other pathways to TCA cycle. This carbon deviation can result in carbon starvation due to the depletion of nonstrucutural carbohydrates. Carbon starvation is one of the main factors commonly associated with forest dieback events under drought conditions^43^. Thus, this complex network of events can result in a higher mortality rate in plants that are experiencing stress for the first time. On the other hand, the existence of a memory mechanism capable of restoring the carbon balance can contribute to the maintenance of the primary productivity of plant communities in environments in which drought occurs as a recurrent stress.

### Metabolic reprogramming, not morphoanatomical changes, are involved in differential acclimation to drought in *D. alata* seedlings

The maintenance of *K*_leaf_, *g*_s_ and physiological processes such as photosynthesis and respiration in 3D plants involved profound reprogramming of cellular metabolism. This contrasting drought response pattern between plants exposed to one or three drought cycles was further revealed by our PCA analysis, with 3D plants showing specific patterns of drought acclimation, such as osmotic adjustment and antioxidant and secondary metabolism activation, while cell damage traits, such as H_2_O_2_ and membrane damage, were strongly associated with 1D plants (Figs. 6, 10). It is important to note that this deep metabolic reprogramming observed in 3D plants was not accompanied by morpho-anatomical changes (Figs. 2, 3). In fact, the only anatomical feature that changed in response to water restriction was the increase in vein density. However, since significant differences were not observed in this trait between 1 and 3D plants, the changes in vein density in relation to control plants cannot explain the differential drought acclimation between the drought treatments. Thus, even considering that changes in vein density may have contributed to the increase plant's fitness, it was not a differential response after recurrent stress and, therefore, it is not characterized as a memory mechanism. This result corroborates the findings of previous studies^23^ which emphasize that stress memory is related to changes in transcriptional and metabolic levels, not to morphoanatomical adjustments. The existence of a drought memory mechanism based on biochemical changes, and not on morphoanatomical modifications, allows plants to acclimatize more quickly and with less energy waste to fluctuating climatic conditions, such as the periodic drought events in the Cerrado. It is also important to emphasize that basal traits, such as morpho-anatomical traits, tend to be more conserved and less responsive to the environment in short term. In contrast, the final resultant traits (physiological/biochemical traits) are usually more integrative and are better to describe the response of plants to environmental factor^44,45^. Lastly, this response pattern can be related with the environment since morphological plasticity has a high cost and will be pre-eminent in plants of resource-rich productive habitats. In plants growing in unfavorable habitats, such as Cerrado, the cellular acclimation appears to be a more important component of the homeostatic mechanisms maintaining tissue viability^46^. Together, these factors reinforce the caution needed to choose traits to describe response of plants to environmental factors.

The absence of cell damage indicators in plants exposed to recurrent water restriction cycles can be explained, at least partially, by the increment in antioxidant system activation, particularly by the higher activity of the enzymes superoxide dismutase (SOD), peroxidase (POX) and glutathione reductase (GR) (Fig. 7), in relation to 1D plants. This set of responses demonstrates the superior ability to detoxify ROS in 3D plants. It is also possible that lower membrane damage in 3D plants is associated to the lipid remodeling in response to drought stress^47,48^, evidenced by the increased levels of palmitic acid in this treatment when compared to 1D plants. Palmitic acid is the main fatty acid present in phospholipids constituents of the plasma membrane and increments in its concentration suggest a reduction in the unsaturation degree of plant membranes^48^. This alters the stability of cell membranes and reduces the occurrence of lipid peroxidation by ROS, given that this process is fundamentally linked to the existence of polyunsaturated fatty acids^49^. Thus, the increase in the levels of palmitic acid, in association with the higher activity of antioxidant enzymes, may have allowed 3D plants to maintain lower levels of electrolyte leakage.

In addition to the changes observed in the cell’s antioxidant machinery, the differential acclimation in 3D plants also required rearrangements in primary and secondary metabolism. Concerning the processes of primary metabolism, photorespiration apparently increased in plants subject to recurrent drought cycles, as evidenced by the increase in glycine levels and apparent glycine/serine ratio (Fig. 8). Recent studies have pointed out that increases in photorespiratory rate represent an important physiological adjustment in plants exposed to drought^40,50^, since under these conditions photorespiration can act as a drain for the excess energy absorbed, thus reducing the formation of ROS and the occurrence of damages to cell structures^39^. In this process, two glycine molecules are converted into a molecule of serine, NH_3_, and CO_2_^40^; for this reason, high values of the glycine/serine ratio have been suggested as the best biochemical parameter to monitor the changes in the photorespiratory activity. In 1D plants, on the other hand, the inability to increase the photorespiratory rate, associated with lower carbon assimilation and low antioxidant system activity, may have contributed to the accumulation of H_2_O_2_ (Figs. 6, 7).

Changes in carbon allocation patterns were not restricted to the altered flux of molecules for photorespiration. On the contrary, differential carbon allocation was an important strategy for drought acclimation in 3D plants, with carbon deviation being observed to supply the production of defense molecules, like the ones involved in osmotic adjustment. The osmotic adjustment is the result of the active accumulation of solute by the plants, which increases water uptake ability, being an important adaptive response of plants to drought conditions^51^. In 3D plants, the metabolic pathway was shifted to osmolyte production, evidencing the occurrence of osmotic adjustment, with increases in the levels of proline, GABA, threonic acid (precursor of threarate, an important compatible solute) and soluble sugars, in comparison to 1D plants. These results explain why plants exposed to recurrent dehydration cycles, despite having greater stomatal conductance and therefore lose more water, were able to maintain their physiological processes. The higher production and accumulation of metabolites associated with osmotic adjustment may also explain the maintenance of *K*_leaf_ in 3D plants, since the maintenance of turgor pressure, mediated by solute accumulation, in addition to allow for higher water uptake, also decreases the leaf hydraulic vulnerability^52^.

Still concerning the changes in carbon allocation, the PCA analysis also showed the carbon deviation from the primary to the secondary metabolism in 3D plants, with higher levels of tryptophan, benzoic acid and nicotinic acid being observed in this treatment (Fig. 8). Tryptophan is a secondary metabolite precursor in plants, synthesized through the shikimic acid pathway, and the increase in its concentration has been suggested as one of the most important characteristics in the response of plants to stress conditions^53^. One secondary metabolite that can be produced from tryptophan is nicotinic acid^54^, the level of which was also higher in 3D plants when compared to 1D plants. Higher levels of nicotinic acid occur as a result of signaling cascades in response to various stressors, such as high temperatures^55^, salinity^56^ and hypoxia^57^, and the accumulation of this metabolite induces several defense responses, such as glutathione synthesis^58^. Finally, benzoic acid, the level of which increased in 3D plants, is a secondary metabolite belonging to the polyphenolic compound class and, in addition to having an antioxidant activity^59^, is the precursor for the synthesis of salicylic acid, an essential hormone in the tolerance of plants to drought^60^. Indeed, it has already been shown that both the application of benzoic acid and salicylic acid increases the tolerance of plants to drought^60–62^.

When analyzed together, the data obtained in the present study allow us to conclude that the recurrent exposure of *D. alata* seedlings to drought cycles resulted in less sensitivity to stress, suggesting the existence of a drought memory mechanism. The greater drought tolerance in 3D plants was evidenced by the maintenance, at normal levels, of essential processes for plant survival, such as photosynthesis, respiration, and hydraulic conductivity. This increase in tolerance, however, involved much more than punctual changes; in fact, this differential acclimation to drought was the result of orchestrated changes in several metabolic pathways, involving differential carbon allocation and the reprogramming and coordination of primary, secondary and antioxidant metabolism. The mechanism of differential acclimation in plants of *D. alata* is probably correlated with the evolutionary history of the species and is, therefore, a reflex of the environment in which this species evolved, the Cerrado, a domain where intense and recurring drought events are intrinsic characteristics. Thus, the characterization of the existence of memory to stress at the domain level represents the next step for studies of ecophysiology and conservation, with important implications for reforestation programs, species conservation and even for the prognosis of the impact of drought events and climatic changes on the biodiversity of environments with specific characteristics.

## Materials and methods

The experiment was conducted at the Ecophysiology and Plant Production Laboratory of the Instituto Federal de Educação, Ciência e Tecnologia Goiano, Campus Rio Verde. *D. alata* seedlings, which were grown from seeds collected in Brazilian Cerrado, were obtained from a local forest nursery and cultivated in 5-L pots containing a mixture of uncultivated soil and sand (2:1, v/v), inside a greenhouse. The plants were irrigated and fertilized as needed, and no restriction to root development was observed at the end of the experiment. After an initial acclimation period (30 days), the plants were submitted to the following treatments: (i)* control* (CT): plants irrigated continuously, with soil moisture maintained close to the field capacity; (ii)* one drought cycle* (1D): plants submitted to a single drought cycle; and (iii)* three drought cycles* (3D): plants submitted to three *consecutive* drought cycles. Each drought cycle consisted of a dehydration phase until the soil moisture reached 25% of available water in relation to the field capacity, with the plants being maintained in these conditions for 6 days; and a recovery phase, during which the plants were irrigated and maintained in field capacity during 15 days. At the end of the 15 days, the plants were evaluated with a gas exchange analyzer to evaluate the liquid carbon assimilation rate (*A*), stomatal conductance (*g*_*s*_) and internal CO_2_ concentration (*Ci*). The evaluation of these traits was carried out as described in item 2.4 (gas exchange). The data obtained from this evaluation demonstrated that 15 days were sufficient to reestablish the carbon assimilation, a key parameter that reflects the plant’s fitness (Supplementary Fig. S1). Based on these results, the plants were considered recovered and a new drought cycle was applied. Plants submitted to one drought cycle remained irrigated while the plants of the 3D treatment were submitted to the first two drought cycles. Thus, the single drought cycle of treatment 1D and the third drought cycle of treatment 3D were applied concomitantly, in plants of the same age and same growth stage. The determination of field capacity was carried out through a soil water retention curve^23^. All the samplings and measurements described below were conducted in the youngest, fully expanded leaves.

### Water relations

The predawn water potential (Ѱ_pd_) was determined with a Scholander type pressure chamber at predawn (04h30–05h30) and expressed as a percentage of the control plants. The determination of the water content in the plant tissues was performed through the weight difference between the fresh and dry material; leaf hydraulic conductivity (*K*_leaf_) was measured using the evaporative flux method and the data were normalized by the leaf area (Brodribb and Holbrook^63^; Simonin et al*.*^64^).

### Morphological analysis

At the end of the experiment, the plants were harvested and segmented into aerial and root parts. The leaves were photographed with a scale and the total leaf area was measured using an image analysis software (ImageJ-National Institutes of Health). The plant material was then oven-dried at 65 °C for 72 h, after which the aerial part dry matter (ADM) and root dry matter (RDM) were determined. The specific leaf area (SLA) was computed as the leaf area per unit of leaf dry mass^65^.

### Leaf anatomy

The epidermis-imprinting technique was used to determine the stomatal density (SD), stomatal index (SI) and guard cell length (L). For these analyses, thirty fields of 0.171 mm^2^ were randomly chosen in microscopic images, and the determination of the SD, SI and the length of the guard cell (L) were performed using image software^31^. The theoretical maximum stomatal conductance (*g*_wmax_) was calculated based on these data, as proposed previously^66^.

To analyze the vein density (VD), fragments of the central part of the leaf blade were preserved in FAA and used in the clarification process, by immersing the leaf samples in 5% sodium hydroxide (NaOH) until the tissue became transparent (48 h). The leaf fragments were then washed several times in distilled water and submitted to a series of ethanolic dehydration (30%, 50%, 70%, and 100%) with subsequent stained with Safranin and Fast Green 1%^67^. Then, the slides were observed at 20 × magnification with the aid of a light microscope (model AX70TRF; Olympus Optical) equipped with the U‐Photo system. The vein density was calculated as the sum of the vein lengths divided by the total image area^68^, using the image analysis program described above.

### Physiological traits

To determine the concentration of chlorophyll *a* and *b*, approximately 0.3 g of fresh material was macerated in liquid nitrogen and subjected to hot ethanol extraction. The absorbance was read at the wavelengths of 665 and 645 nm, to determine the chlorophylls *a* and *b*, respectively^69^.

Leaf gas exchange and chlorophyll *a* fluorescence were measured simultaneously with an open‐flow infrared gas exchange analyzer system equipped with a leaf chamber fluorometer (LI‐6800; Li‐Cor). The light-saturated net CO_2_ assimilation rate (*A*), the stomatal conductance (*g*_*s*_), the substomatal CO_2_ concentration (*Ci*) and the transpiration rate (*E*) were determined on attached, fully expanded leaves. The water use efficiency was estimated by the ratio between the net carbon assimilation rate and the leaf transpiration (*A*/*E*). The Rubisco maximum carboxylation rate (V_cmax_) was estimated using the one-point method^70^. Environmental conditions in the leaf chamber consisted of a photosynthetic photon flux density of 1000 µmol m^−2^ s^−1^, a vapor pressure deficit of 1.0–1.5 kPa, an air temperature of 25 °C and an ambient CO_2_ concentration of 400 µmol mol^−1^ air. The dark respiration (*R*_*N*_) was measured before dawn using the infrared gas analyzer mentioned above.

Analysis of the minimal fluorescence (*F*_*0*_) was performed before the dawn period via excitation of the leaf tissues with a modulated red light of low intensity (0.03‐µmol photon m^−2^ s^−1^). To obtain maximum fluorescence (*F*_*m*_), saturation pulses of approximately 8000‐µmol photon m^−2^ s^−1^ were applied for 0.8 s. The variable fluorescence (*F*_*v*_) was determined by the difference between *F*_*0*_ and *F*_*m*_, and the potential quantum yield of photosystem II was calculated from these values (*F*_v_/*F*_m)_^71^.

### Evaluation of hydrogen peroxide concentration and cell damage

For determining the concentration of hydrogen peroxide (H_2_O_2_), samples of 0.3 g of leaves and roots were homogenized in the extraction medium and centrifuged at 10,000 × *g* for 15 min at 4 °C^72^. Aliquots of 50 µL of supernatant were added to the reaction medium containing 100 µM FeNH_4_SO_4_, 25 mM sulfuric acid, 250 µM xylenol orange and 100 mM sorbitol^73^. Samples were kept in the dark for 30 min, and the absorbance was determined at 560 nm. H_2_O_2_ concentrations were estimated based on a calibration curve.

The damage to cell membranes was characterized by the electrolyte release rate from ten leaf discs with 1 cm in diameter immersed in 10 mL of deionized water and maintained at room temperature for 8 h. The initial conductivity (*C*1) was measured with a conductivity instrument. The samples were then transferred to a conventional oven at 90 °C for 1 h to induce maximum leakage. After cooling down at room temperature, electrolyte conductivity (*C*2) was measured, and the relative electrical conductivity (*C*%) was calculated as: (*C*1/*C*2) × 100^74^.

### Determination of antioxidant enzyme activity

To assess the activity of the antioxidants enzymes, 0.3 g of fresh leaves were homogenized in the extraction medium (0.1 M potassium phosphate buffer, pH 6.8, 0.1 mM EDTA, 1 mM phenylmethanesulfonyl fluoride, and 1% polyvinylpyrrolidone)^75^. The homogenate was centrifuged at 12,000 × *g* for 15 min at 4 °C. The resulting supernatant was used as a crude extract for the assessment of superoxide dismutase (SOD), peroxidase (POX), ascorbate peroxidase (APX), catalase (CAT) and glutathione reductase (GR) activities. The SOD activity (SOD, EC 1.15.1.1) was measured as the inhibition of p-nitro tetrazolium photoreduction^76^. The enzymatic activity was expressed in SOD units corresponding to the amount of enzyme required to inhibit 50% of the p-nitro blue tetrazolium photoreduction. POX activity (POX, EC 1.11.1.7) was assessed through the production rate of purpurogallin at 420 nm with a molar extinction coefficient of 2.47 mmol^−1^ L cm^−1^^77^. The enzymatic activity was expressed in micromoles purpurogallin min^−1^ g^−1^ fresh weight (FW). The APX activity (APX, EC 1.11.1.11) was assessed as the rate of ascorbate oxidation at 290 nm^77^ using a molar extinction coefficient of 2.8 mmol^−1^ L cm^−1^. The enzymatic activity was expressed in micromoles ascorbate min^−1^ g^−1^ FW. The CAT activity (CAT, EC 1.11.1.6) was estimated through the decomposition of H_2_O_2_ during the first minute of the reaction at 240 nm^78^ using a molar extinction coefficient of 36 mol^−1^ L cm^−1^. The enzymatic activity was expressed in micromoles H_2_O_2_ min^−1^ g^−1^ FW. The GR activity (GR, EC 1.8.1.7) was determined by monitoring the oxidation of NADPH as visualized by a decrease in absorbance at 340 nm during the first minute of the reaction and using a molar extinction coefficient of 6.22 mol^−1^ L cm^−1^^79^.

### Metabolite profiling

Metabolite extraction was performed by grinding the lyophilized leaf tissues (approximately 75 mg) with the addition of the appropriate extraction buffers. To obtain a broad overview of the major pathways of central metabolism, an established gas chromatography-mass spectrometry (GC–MS-based) metabolite profiling method^80^ was used to quantify the relative metabolite levels in response to the imposed treatments. The extraction, derivatization, standard addition, and sample injection were performed exactly as described previously^81^. Chromatograms and mass spectra were evaluated by using Chroma TOF 1.0 (Leco, https://www.leco.com/) and TagFinder 4.0 software. Metabolite identification was manually supervised using the mass spectral and retention index collection of the Golm Metabolome Database in comparison with database entries of authentic standards^82,83^. Peak heights of the mass fragments were normalized on the basis of the dry weight of the sample and the added amount of an internal standard (ribitol). Data were normalized with respect to the mean response calculated for the control plants (to allow statistical assessment, individual plants from this set were normalized in the same way).

### Statistical analyses

All experiments were conducted in a completely randomized experimental design with eight repetitions in each treatment. Each repetition was composed by an individual. Five completely expanded young leaves were collected in each repetition to perform the analyzes. Results were analyzed using analysis of variance (ANOVA) and differences between means were revealed by Student Newman Keuls test (SNK) (*P* ≤ 0.05). To study the relationships between variables, a principal component analysis (PCA) was used. The PCA was performed using the normalized data because these data preserved the distance between the measured variables. Treatments with a similar behavior were clustered using a multivariate grouping analysis technique based on average Euclidean distances. Statistical analyzes were performed using SISVAR 5.6^84^ and RStudio^85^.

## Supplementary information

Supplementary Information.
