# Supplementary material for: Evidence of drought memory in Dipteryx alata indicates differential acclimation of plants to savanna conditions
Source: Sci Rep. 2020 Oct 5;10:16455. doi: 10.1038/s41598-020-73423-3 (PMC7536413; doi:10.1038/s41598-020-73423-3)
Supplement: Supplementary file 1 — Supplementary Information. [file 41598_2020_73423_MOESM1_ESM.pdf]

Supplementary Material

Evidence of Drought Memory in *Dipteryx alata* Indicates Differential Acclimation of plants to Savanna Condition

Rauander D.F.B. Alves; Paulo E. Menezes-Silva; Leticia F. Sousa; Lucas Loram-Lourenço; Maria L.F. Silva; Sabrina E.S. Almeida; Fabiano G. Silva; Leonardo P. Souza; Alisdair R. Fernie; Fernanda S. Farnese.

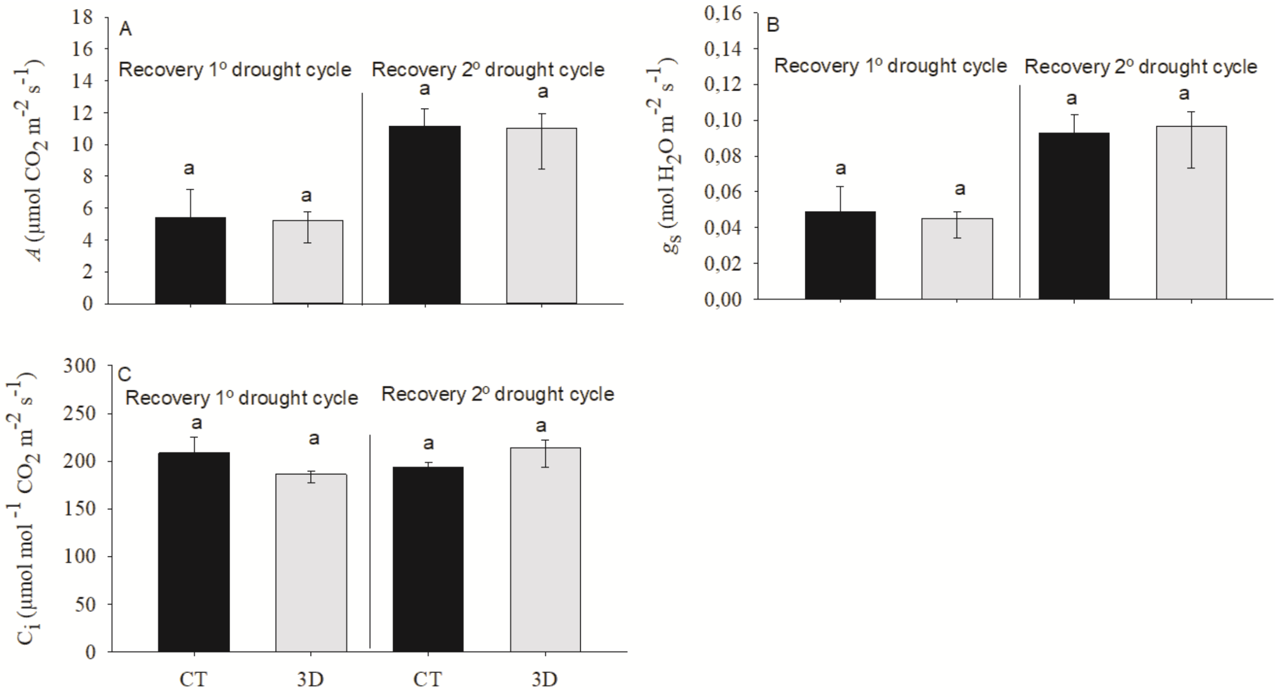

Supplementary Figure S1 - Net carbon assimilation rate (*A*) (A), Stomatal conductance (g_s) (B) and Internal CO_2 concentration (Ci) (C) in *Dipteryx alata* seedlings irrigated for a period of 15 days after the first drought cycle (Recovery 1° drought cycle) and the second drought cycle (Recovery 2° drought cycle). Means followed by the same letter do not differ from each other by the SNK test ($P \leq 0.05$).
